# Long‐Term Elevated CO_2_ Improves Soil Health and Rice Yields in Paddy Fields

**DOI:** 10.1002/advs.202503190

**Published:** 2025-12-07

**Authors:** Fan Jiang, Wenchao Du, Kees Jan van Groenigen, Josep Peñuelas, Scott X. Chang, Lian Song, Chuang Cai, Yu Wang, Lin Deng, Zhengkun Hu, Weishou Shen, Qicong Wu, Haiyang Yu, Ying Yin, Fuxun Ai, Wei Zhou, Dongming Wang, Jianqiang Sun, Xiaoyuan Yan, Renfang Shen, Jiabao Zhang, Hongyan Guo, Chunwu Zhu

**Affiliations:** ^1^ State Key Laboratory of Water Pollution Control and Green Resource Recycling, School of Environment Nanjing University Nanjing 210023 China; ^2^ School of Environment Nanjing Normal University Nanjing 210023 China; ^3^ Department of Geography, Faculty of Environment, Science and Economy University of Exeter Exeter EX4 4QJ UK; ^4^ CSIC, Global Ecology Unit CREAF‐CSIC‐UAB Bellaterra Barcelona 08193 Spain; ^5^ CREAF Cerdanyola del Vallès Barcelona 08193 Spain; ^6^ Department of Renewable Resources University of Alberta Alberta T6G 2R3 Canada; ^7^ State Key Laboratory of Soil and Sustainable Agriculture, Institute of Soil Science Chinese Academy of Sciences Nanjing 211135 China; ^8^ College of Resources and Environmental Sciences Nanjing Agricultural University Nanjing 210095 China; ^9^ School of Environmental Science and Engineering Nanjing University of Information Science and Technology Nanjing 210044 China; ^10^ College of Forestry Shandong Agricultural University Taian 271018 China; ^11^ Key Laboratory of Urban Environment and Health, Institute of Urban Environment Chinese Academy of Sciences Xiamen 361012 China; ^12^ Institute of Agricultural Resources and Regional Planning Chinese Academy of Agricultural Sciences Beijing 100081 China; ^13^ College of Environment Zhejiang University of Technology Hangzhou 310014 China

**Keywords:** CO_2_ fertilization effect, food security, global change, rice production, soil quality

## Abstract

Soil health underpins the productivity and ecosystem functioning of rice paddies, yet its response to elevated atmospheric CO_2_ (eCO_2_) remains poorly understood. Here, soil health responses to eCO_2_ are evaluated using the two longest‐running rice free‐air CO_2_ enrichment experiments, spanning 12 and 15 years. The results show that long‐term eCO_2_ significantly improves soil health, strengthening its capacity to support crop production, water purification, and climate change mitigation. Integration of global observations further indicates that these improvements are widespread and cumulative over time, with paddy soils benefiting more than other terrestrial ecosystems. Consequently, long‐term eCO_2_ exposure tends to enhance rice yield gains, in contrast to the productivity plateau observed in natural ecosystems. These findings provide novel and comprehensive evidence that long‐term eCO_2_ enhances paddy soil health, improving soil multifunctionality and reinforcing the rice CO_2_ fertilization effect.

## Graphical abstract

Combining the two longest‐running rice free‐air CO_2_ enrichment experiments with a global data synthesis, this study demonstrates that long‐term elevated CO_2_ consistently enhances soil health. In rice paddies, this improvement sustains the CO_2_ fertilization effect over decades. Proactive measures to avert potential phosphorus deficiency and soil acidification are essential, allowing healthier soils to further reinforce future food security.

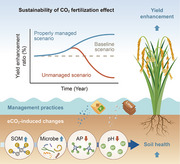

## 1 Introduction

Rice is a staple food for over half of the world's population, which makes its production essential for global food security.^[^ ^1^, ^2^ ^]^ Elevated atmospheric CO_2_ concentrations (eCO_2_) are expected to boost rice yields through the CO_2_ fertilization effect.^[^ ^3^ ^]^ However, this prediction is largely based on short‐term studies, leaving uncertainties regarding the long‐term impacts of eCO_2_ on rice productivity.^[^ ^2^, ^4^ ^]^ This concern is not unwarranted, as studies in other ecosystems suggest that ignoring the effects of long‐term eCO_2_ exposure on soils may lead to overestimates of plant productivity.^[^ ^5^, ^6^, ^7^ ^]^

Given the diverse effects of eCO_2_ on soils, predictions of rice yield responses require consideration of the broader soil context, as focusing on isolated variables may produce contradictory conclusions.^[^ ^8^ ^]^ For instance, prolonged exposure to eCO_2_ generally increases soil organic carbon (SOC),^[^ ^9^, ^10^, ^11^ ^]^ but it can also reduce available phosphorus (AP).^[^ ^12^, ^13^ ^]^ While higher SOC promotes crop yields,^[^ ^14^ ^]^ a simultaneous decline in AP increases the risk of yield reductions due to phosphorus (P) deficiency in rice paddies.^[^ ^12^ ^]^ Thus, evaluating multiple soil properties is necessary to reliably assess the long‐term effect of eCO_2_ on rice productivity.

Soil health assessments integrate key indicators that reflect the capacity of soil to deliver multiple ecosystem services.^[^ ^15^, ^16^ ^]^ The definition of soil health varies by land‐use type, with context‐dependent priorities.^[^ ^17^, ^18^ ^]^ In rice paddies, as agricultural land, the primary concern is the soil's capacity to sustain plant production. However, rice paddies are also key leverage points for climate change mitigation and water quality control, since they are an important source of greenhouse gas (GHG) emissions^[^ ^1^ ^]^ and prone to nutrient leaching from periodic drainage.^[^ ^12^, ^19^ ^]^ Due to disruptions in biogeochemical cycles caused by intensive human activity, rice paddies may rely more on soil health to sustain ecosystem services than other land‐use types.^[^ ^20^ ^]^ A recent study has shown that healthier soils are associated with higher rice yields and greater yield stability.^[^ ^21^ ^]^ However, the impact of eCO_2_ on paddy soil health—and its implications for the long‐term sustainability of CO_2_ fertilization—remains unclear.

Soil microbes, particularly fungi, are integral to soil health,^[^ ^20^, ^22^ ^]^ and their community structure and functions are highly sensitive to long‐term eCO_2_.^[^ ^23^, ^24^, ^25^ ^]^ Although microbial biomass is commonly included in soil health evaluations,^[^ ^18^ ^]^ its effectiveness as a biological indicator is limited due to its “black‐box” nature.^[^ ^22^, ^26^ ^]^ Beyond biomass, the composition and functional roles of microbial communities are crucial for maintaining soil health.^[^ ^18^, ^22^ ^]^ Globally, symbiotic associations with fungi play a key role in governing the responses of the soil‐plant continuum to eCO_2_.^[^ ^27^, ^28^ ^]^ A recent continental‐scale soil health survey in Europe further confirmed that mycorrhizal fungi are critical for sustaining cropland productivity.^[^ ^20^ ^]^ Consequently, understanding how eCO_2_ influences soil fungal functions is essential for predicting changes in rice productivity under future climate conditions.

In this study, we hypothesize that long‐term eCO_2_ exposure alters the sustainability of the CO_2_ fertilization effect by modulating soil health in rice paddies. To test this hypothesis under real‐world conditions, we conducted two of the world's longest‐running rice free‐air CO_2_ enrichment (FACE) experiments in China, spanning 12 and 15 years. The two study sites, Jiangdu FACE (NP_M_; Figure S1a, Supporting Information) and Changshu FACE (NP_H_; Figure S1b, Supporting Information), represent rice paddies with moderate and high nitrogen (N) and P levels, respectively (Figure S2, Supporting Information). Soil health was evaluated using the Cornell Comprehensive Assessment of Soil Health (CASH) protocol, integrating eleven soil properties that capture key ecosystem services, including food production, water quality regulation, and climate change mitigation (Table S1, Supporting Information). To evaluate the representativeness of our findings, we synthesized global observations of soil health responses to eCO_2_ and examined how responses in rice paddies vary from those in other terrestrial ecosystems. Complementary analyses of soil fungal functional shifts were also conducted to provide biotic insights into soil health changes. This study emphasizes the vital role of soil health in sustaining the long‐term CO_2_ fertilization effect, offering guidance for developing a sustainable, climate‐resilient rice production system.

## 2 Results

### 2.1 Impact of eCO_2_ on Paddy Soil Health

**Figure 1:**
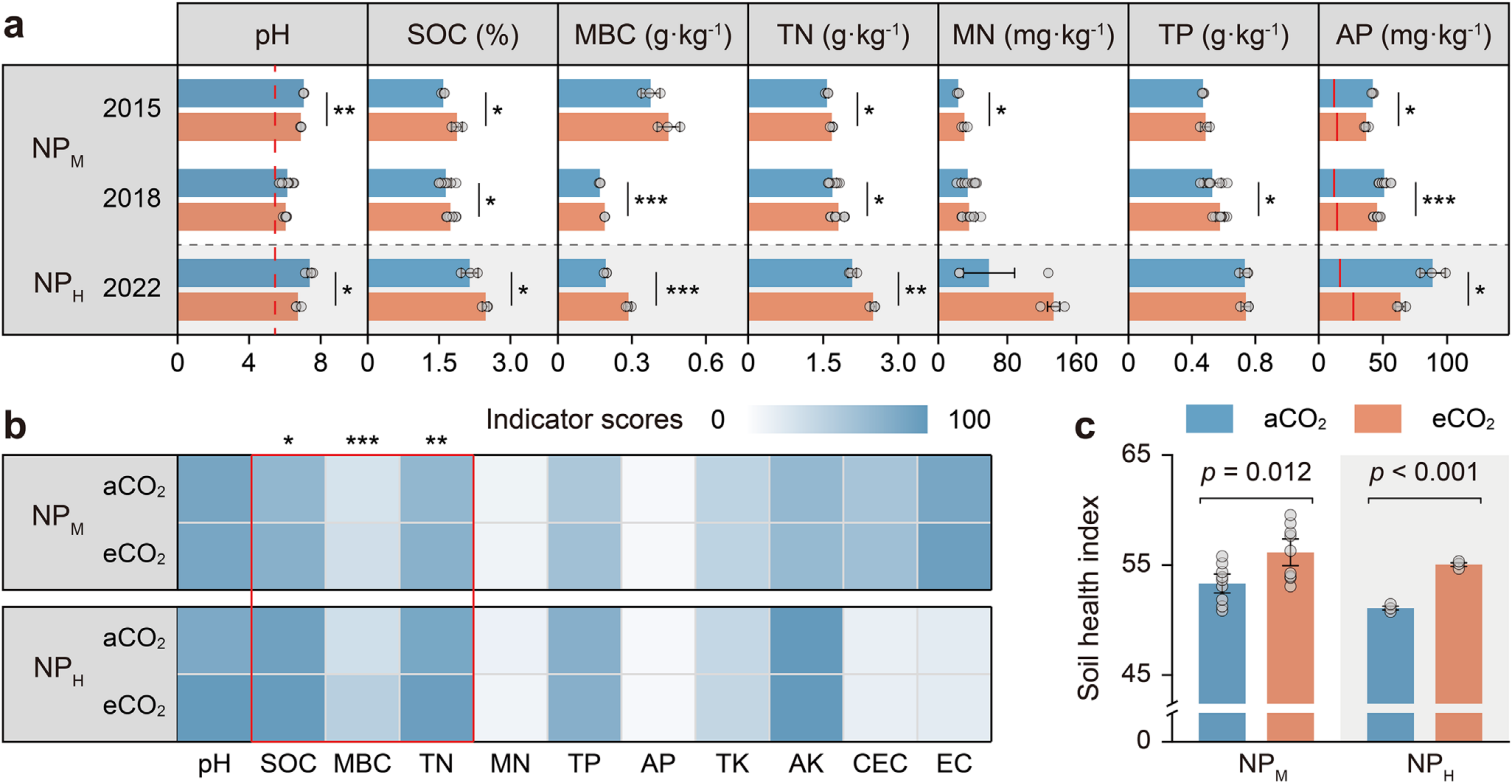
Soil health responses to long‐term eCO_2_ in two Chinese rice FACE experiments. a) Response of soil properties to eCO_2_. b) Changes in soil health indicator scores after the longest eCO_2_ exposure (NP_M_ 2018 and NP_H_ 2022). TK, total potassium; AK, available potassium; CEC, cation exchange capacity; EC, electrical conductivity. c) Soil health index for NP_M_ 2018 and NP_H_ 2022. Data are presented as mean ± SD (*n* = 9 for NP_M_ 2018; *n* = 3 for NP_M_ 2015 and NP_H_ 2022). In a) and c), differences were assessed using Student's *t*‐test for normally distributed data and Mann–Whitney U test otherwise; in b), eCO_2_ effects were identified using generalized linear models (GLMs). *p < 0.05; **p < 0.01; ***p < 0.001. The red dashed and solid lines in a) indicate the pH and AP thresholds for acidity stress (yield ≥ 95% of maximum)^[^ ^29^ ^]^ and P deficiency (Figure S5, Supporting Information), respectively. Supplementary indicators used in b) are shown in Figure S4 (Supporting Information).

Long‐term exposure to eCO_2_ significantly increased SOC, microbial biomass carbon (MBC), and total nitrogen (TN), with the effects being more pronounced at the NP_H_ site (**Figure** **1**a). Although responses varied across years, eCO_2_ increased mineral nitrogen (MN) in 2015 and total phosphorus (TP) in 2018 at the NP_M_ site. By converting soil property measurements into ecologically relevant soil health status (Figures S3,S4, Supporting Information), we found that long‐term eCO_2_ improved SOC, MBC, and TN scores overall in our two study sites (Figure 1b).

As anticipated, eCO_2_ lowered soil pH, yet it remained within a range safe for rice growth (Figure 1a). Likewise, conservative estimates of P balance showed that AP under eCO_2_ stayed well above thresholds for P deficiency (Figure S5, Supporting Information), and thus posed no adverse effect on grain P content (Figure S6, Supporting Information). Indeed, as P pollution risk declined, AP scores tended to increase (*p* = 0.058). Collectively, our findings suggest that the soil health index—an integrated measure of multiple soil ecosystem services—improved under long‐term eCO_2_ (Figure 1c).

### 2.2 Consistency and Variability of Soil Health Responses to eCO_2_

**Figure 2:**
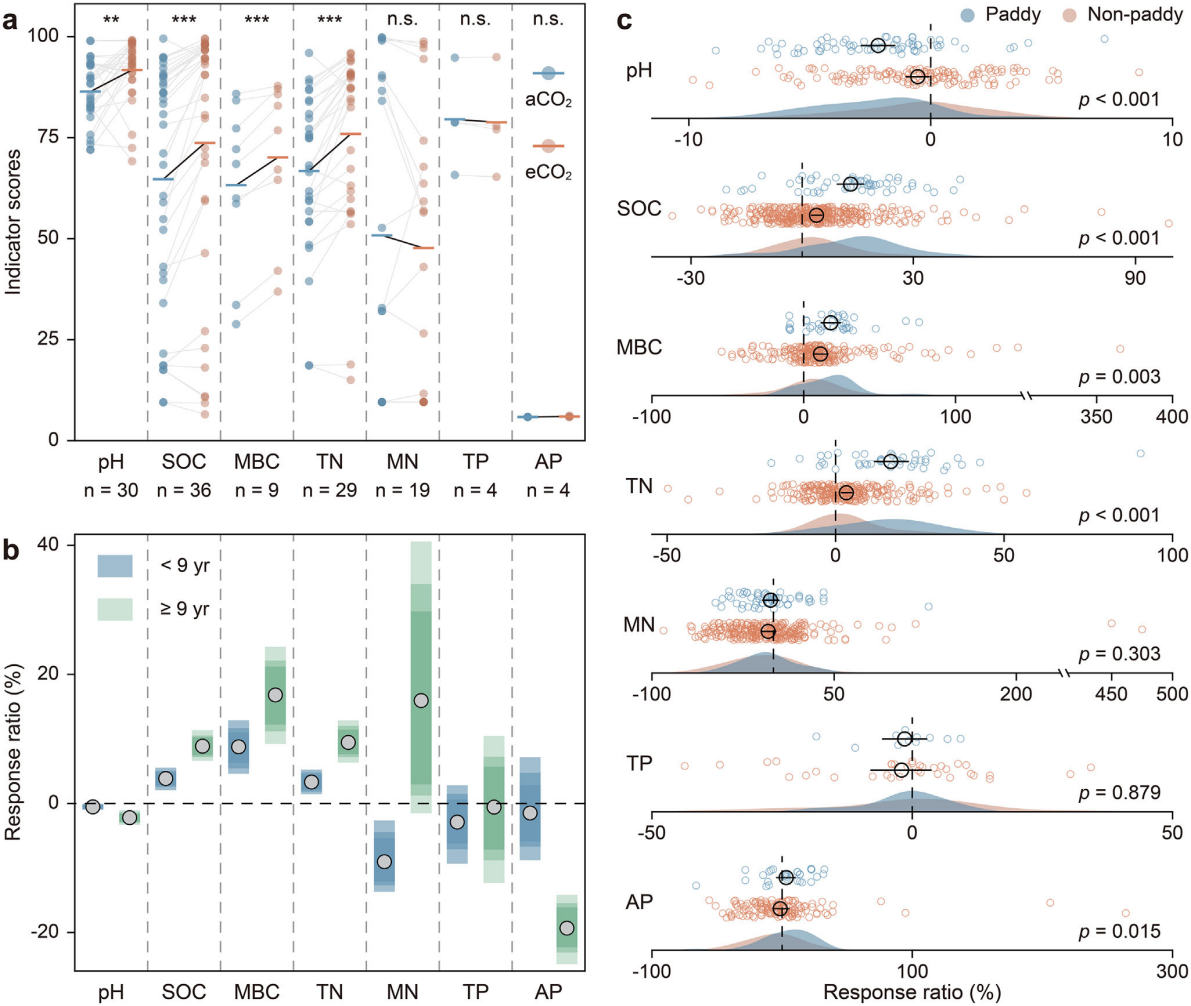
Response patterns of soil health to eCO_2_ based on a global dataset. a) Responses of indicator scores to eCO_2_ in Chinese rice paddies. b) Differences between short‐ and long‐term studies across global terrestrial ecosystems. c) Variability of indicator responses in paddy and non‐paddy soils. Data are available in Data S1 and S2 (Supporting Information). In a), data from this study were excluded to ensure objectivity; thick horizontal lines indicate group means; paired‐sample *t*‐tests were used for significance testing (**p < 0.01; ***p < 0.001). In b), circles represent means, and colored bars represent 95%, 85%, and 75% confidence intervals (lighter colors correspond to higher confidence). In c), black circles and error bars denote the mean ± 95% CI, and significance was evaluated using the Mann–Whitney U test.

To assess the broader applicability of our findings, we synthesized 779 observations from 253 eCO_2_ experiments worldwide, focusing on the responses of soil health indicators to eCO_2_ (Figure S7, Supporting Information). From these records, we first extracted field‐based data from Chinese rice paddies and incorporated them into our assessment framework. Consistent with our FACE results, indicator scores for SOC, MBC, and TN in rice paddies increased under eCO_2_ (**Figure** **2**a). These gains in soil health tended to become more pronounced with prolonged eCO_2_ exposure (Figure S8, Supporting Information). Notably, given the relatively high baseline soil pH at these sites (7.09 ± 0.56), eCO_2_‐induced acidification increased rather than decreased the pH indicator scores, with declines occurring only when pH fell below 6.67 (Figure S3k, Supporting Information).

Across terrestrial ecosystems, eCO_2_ consistently decreased soil pH while increasing SOC, MBC, and TN, with effect sizes strengthening over the duration of CO_2_ enrichment (Figure 2b; Figure S9a–d, Supporting Information). Nutrient availability, however, exhibited time‐dependent trajectories. In short‐term experiments (<9 years), MN responses were negative and AP responses remained neutral; over longer durations (≥9 years), MN responses shifted toward neutral or positive, whereas AP responses turned increasingly negative. The inflection points of this directional shift occurred after ≈7.2 years for MN and 2.4 years for AP under eCO_2_ exposure (Figure S9e,g, Supporting Information). Soil TP remained largely unaffected by eCO_2_, except under strong microbial P limitation, where positive responses emerged (Figure S10, Supporting Information).

Soil responses to eCO_2_ varied substantially in magnitude across ecosystem types. In non‐paddy ecosystems (uplands, grasslands, and forests), declines in soil pH were relatively small, whereas rice paddies exhibited stronger increases in SOC, MBC, and TN, along with smaller reductions in AP (Figure 2c). Nonetheless, the direction of soil health responses was largely consistent, indicating that the beneficial effects of long‐term eCO_2_ are not confined to our study sites but apply broadly across terrestrial ecosystems. Importantly, because SOC, MBC, and TN, the key drivers of soil health enhancement (Figure 1b), increase most strongly in rice paddies, this land‐use type appears to benefit most from rising atmospheric CO_2_.

### 2.3 Fungi as a Biotic Component of Soil Health

**Figure 3:**
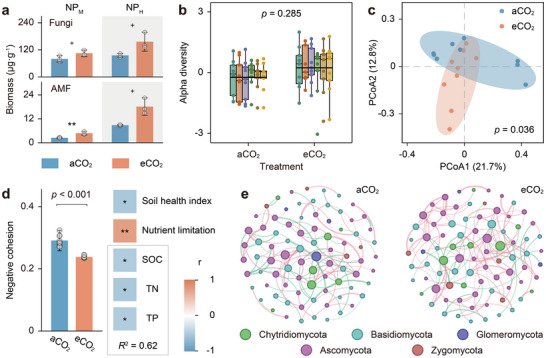
Soil fungal responses to long‐term eCO_2_ in two Chinese rice FACE experiments. a) Biomass of fungi and AMF. b) *α*‐diversity of fungal communities. c) Fungal community composition. d) Interspecific competition and its relationship with nutrient limitation. e) Co‐occurrence networks among fungal genera. Soil samples for fungal biomass were collected from NP_M_ in 2018 and NP_H_ in 2022 (one mixed sample per plot, *n* = 3), and for community analysis from NP_M_ in 2018 (*n* = 9). In a) and d), data are presented as mean ± SD; significance was assessed by Student's *t*‐test (^+^ *p* < 0.10; **p < 0.01). Box plots in b) show *z*‐standardized *α*‐diversity indices (see Experimental Section), with black line indicating means. In b) and c), overall differences in diversity and community structure were tested by PERMANOVA. In d), hierarchical partitioning identified the relative contribution of nutrient supply to the variation in interspecific interactions. In e), red and green edges represent positive and negative correlations, respectively (Spearman's *ρ* > 0.8, *p* < 0.01), with edge width and node size scaled to correlation strength and connectivity.

Soil fungal biomass tended to increase under long‐term eCO_2_, particularly at the NP_M_ site, where arbuscular mycorrhizal fungi (AMF) nearly doubled (**Figure** **3**a). Beyond mycorrhizal taxa, fungi involved in decomposing recalcitrant substrates such as cellulose and chitin were also enriched (Figures S11,S12, Supporting Information). Although these functional shifts were not directly incorporated into the soil health indices due to methodological limitations, our results clearly demonstrate that long‐term eCO_2_ enhances fungal capacity to facilitate nutrient cycling and plant growth, thereby supporting improved soil health.

Fungal responses to eCO_2_ were reflected in changes in community structure rather than diversity (Figure 3b,c), characterized by reduced interspecific competition, which was closely associated with the alleviation of nutrient limitation accompanying improved soil health (Figure 3d). Hierarchical partitioning analysis showed that increased supplies of carbon (C), N, and P together explained 62% of the observed variation in interspecific interactions. Metabolomic profiling confirmed that soils under eCO_2_ contained higher levels of bioavailable small molecules, particularly carbohydrates and fatty acids (Figure S13, Supporting Information). Consistent with these patterns, eCO_2_ strengthened positive interspecific correlations (Figure 3e; Table S2, Supporting Information) and increased stochasticity in community assembly (Figure S14, Supporting Information), both indicative of reduced environmental stress.

### 2.4 Relationship Between Soil Health and Rice Yield

**Figure 4:**
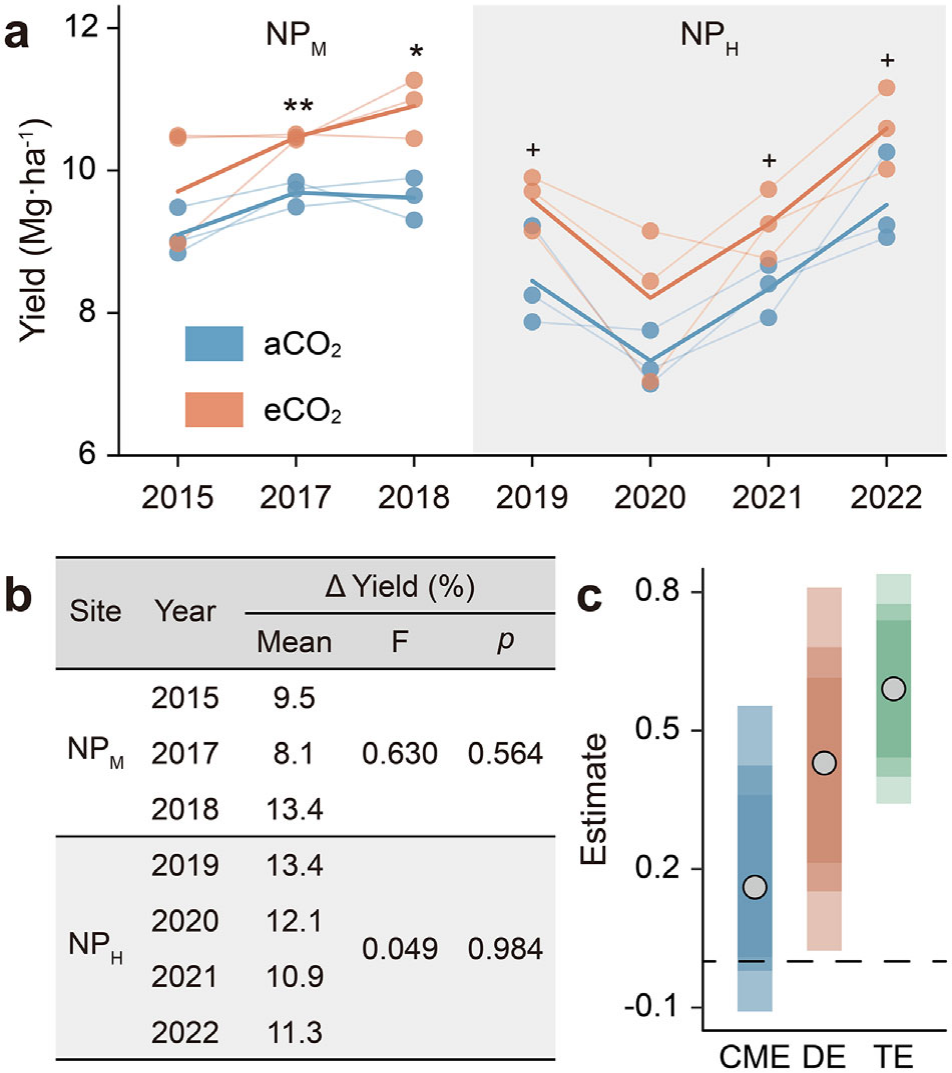
Rice yield responses to long‐term eCO_2_ in two Chinese rice FACE experiments. a) Temporal trends in rice yield. b) Interannual variation in yield increase. c) Causal mediation analysis of eCO_2_ effects on yield. In a), light lines show yield per plot (*n* = 3) and dark lines indicate the mean; differences were tested by Student's t‐test (^+^ *p* < 0.10; *p < 0.05; **p < 0.01). In b), the effect of duration on yield increase was tested using one‐way ANOVA. In c), the total effect of eCO_2_ on yield (TE) is decomposed into the direct effect (DE) of CO_2_ fertilization and the soil health‐mediated effect (causal mediation effect, CME); circles show point estimates, and colored bars represent 95%, 85%, and 75% confidence intervals (lighter colors indicate higher confidence).

Across both experimental sites, eCO_2_ increased rice yield by an average of 11.2% (**Figure** **4**a). Importantly, this CO_2_ fertilization effect persisted over time (Figure 4b), and eCO_2_ did not decrease N and P uptake in rice grains (Figure S6, Supporting Information). In line with our expectations, the overall soil health index showed a stronger correlation with rice yield than any individual soil property (Figure S15, Supporting Information). Specifically, the correlation between soil health and rice yield was 5.01‐fold higher than the average correlation with individual soil properties and 5.79‐fold higher than the average correlation with individual indicator scores (Table S3, Supporting Information), confirming the validity of our soil health assessment framework.

Causal mediation analysis indicated that the primary driver of the yield increase was the direct CO_2_ fertilization effect (i.e., stimulation of photosynthesis). Nevertheless, improvements in soil health also contributed, bringing the total positive effect of eCO_2_ on rice yield to 1.42‐fold via soil health‐mediated indirect effects (Figure 4c).

## 3 Discussion

### 3.1 Commonalities and Divergences in Soil Health Responses to eCO_2_

Our study provides compelling evidence that long‐term eCO_2_ exposure improves soil health in rice paddies (Figures 1c and 2a). This improvement is driven by increases in SOC, TN, and MBC (Figure 1b), which are broadly consistent across terrestrial ecosystems (Figure 2b) and accumulate with prolonged eCO_2_ exposure (Figures S8 and S9b–d, Supporting Information). SOC accumulation mitigates rising atmospheric CO_2_,^[^ ^18^, ^20^ ^]^ enhances plant productivity,^[^ ^14^ ^]^ and improves soil pollutant retention, thereby reducing nutrient and toxin runoff into water.^[^ ^18^ ^]^ The concurrent rise in TN expands long‐term nutrient reserves, thus reducing dependence on N fertilizers in crop production.^[^ ^15^, ^30^ ^]^ As the living component of soil organic matter (SOM), microbial biomass serves as a key vehicle for cycling biophilic nutrients and thus plays a central role in maintaining soil fertility.^[^ ^20^, ^23^, ^31^ ^]^ Collectively, these changes signify broad improvements in soil ecosystem services that underpin agricultural productivity, water quality regulation, and climate change mitigation (Table S1, Supporting Information).

Across terrestrial ecosystems, soil responses to eCO_2_ generally follow similar underlying mechanisms.^[^ ^32^ ^]^ Within the soil‐plant continuum, eCO_2_ initially stimulates plant growth, increasing plant residue^[^ ^27^, ^33^ ^]^ and rhizodeposit^[^ ^8^, ^32^ ^]^ inputs that deliver additional C, along with N and P, to the soil.^[^ ^6^, ^13^ ^]^ Subsequently, distinct biological processes govern the fate of N and P pools.^[^ ^34^ ^]^ Enhanced microbial activity under eCO_2_ typically promotes N sequestration, as greater C availability favors biological N fixation over N mineralization.^[^ ^9^, ^10^ ^]^ By contrast, the net microbial effect on P cycling is highly context‐dependent. In croplands, soil microbes are seldom constrained by P;^[^ ^35^ ^]^ thus, surplus P tends to undergo mineralization rather than be retained.^[^ ^12^, ^36^ ^]^ This pattern aligns with our FACE results, in which elevated C inputs suppressed TP accumulation (Figures S16 and S17, Supporting Information). As microbial P limitation intensifies, however, P assimilation gradually dominates, resulting in substantial TP increases in P‐limited natural ecosystems (Figure S10, Supporting Information).^[^ ^6^, ^34^ ^]^ Consequently, on a global scale, the linkage between soil C and P dynamics under eCO_2_ is highly variable, whereas the C–N response remains tightly coupled (Figure S18, Supporting Information). Although not all additional C and nutrients are retained, eCO_2_ consistently enhances their fluxes into the soil,^[^ ^33^ ^]^ thereby increasing resource availability for microbial growth and leading to higher MBC.^[^ ^11^ ^]^

Despite these overarching similarities, rice paddies derive greater benefits from eCO_2_ (Figure 2c). This advantage stems from their waterlogged, anaerobic conditions, which suppress microbial decomposition of plant residues and thereby preserve SOC and TN.^[^ ^37^ ^]^ Moreover, paddy soils are typically enriched in metal oxides, which promote long‐term SOM stabilization through mineral–organic associations.^[^ ^38^, ^39^ ^]^ In contrast, in non‐paddy ecosystems, a considerable portion of the additional C gained under eCO_2_ is offset by the priming effect (i.e., accelerated SOM decomposition induced by labile C inputs),^[^ ^40^, ^41^ ^]^ even for fractions otherwise protected by soil minerals.^[^ ^42^ ^]^ Regarding MBC accumulation, the unbalanced soil stoichiometry caused by intensive inorganic fertilization in croplands often places microbes under strong C limitation,^[^ ^35^ ^]^ substantially enhancing their carbon use efficiency (CUE).^[^ ^43^, ^44^ ^]^ Conversely, in nutrient‐limited natural ecosystems, soil microbes are unable to assimilate surplus C and instead release it via respiration.^[^ ^40^, ^45^ ^]^ Consequently, both upland and paddy soils exhibit strong and comparable MBC responses relative to non‐cropland soils (Figure S19, Supporting Information). Such CUE variations are also likely to amplify long‐term heterogeneity in SOC and TN responses to eCO_2_ across ecosystems, as higher CUE generally promotes microbial necromass formation and, in turn, enhanced SOM stabilization over extended timescales.^[^ ^31^, ^38^ ^]^

### 3.2 Contribution of Fungi to Soil Health Improvement

Soil microbes respond to eCO_2_ primarily through plant‐mediated mechanisms, with functional shifts in microbial communities feeding back to plant performance.^[^ ^23^, ^27^, ^33^ ^]^ In this study, increased fungal biomass (Figure 3a), reduced environmental filtering (Figure 3d; Figure S14, Supporting Information),^[^ ^46^ ^]^ and strengthened cooperative interspecific interactions (Figure 3e)^[^ ^47^ ^]^ collectively indicate a relaxation of microbial growth constraints. These patterns largely arise from the expansion of bioavailable small molecules and nutrient pools accompanying improved soil health (Figure 3d; Figure S13, Supporting Information), supplying abundant resources to sustain microbial metabolism.

The alleviation of environmental stress on fungal communities under eCO_2_ is evident in the increased abundance of both saprotrophic fungi and AMF (Figure 3a; Figures S11,S12, Supporting Information). Saprotrophic fungi accelerate soil nutrient cycling, particularly N mineralization (Figures S20,S21, Supporting Information), helping sustain the CO_2_ fertilization effect under long‐term eCO_2_.^[^ ^8^, ^48^ ^]^ Meanwhile, AMF—the most widespread mycorrhizal symbionts—improve plant nutrient acquisition and promote cropland productivity.^[^ ^20^, ^28^ ^]^ Collectively, these positive shifts in interspecific interactions, driven by improved soil health, promote the proliferation of beneficial fungi, creating a positive feedback that further improves soil health under eCO_2_.

### 3.3 Soil Health and the Sustainability of the CO_2_ Fertilization Effect

**Figure 5:**
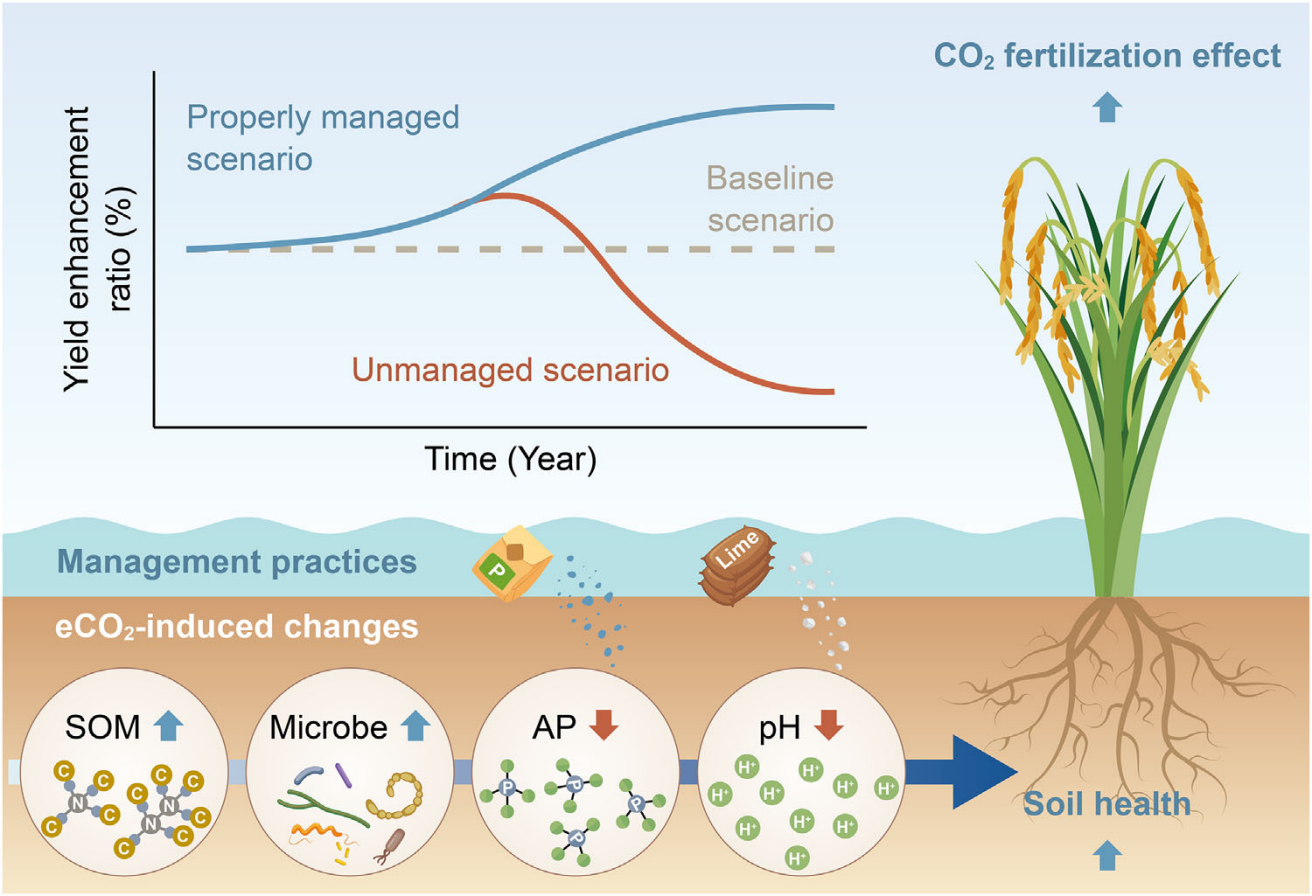
Schematic of soil health‐mediated sustainability of the rice CO_2_ fertilization effect. Baseline scenario: considers only the stimulation of plant photosynthesis by eCO_2_. Unmanaged scenario: accounts for soil health changes under long‐term eCO_2_, without interventions to mitigate soil acidification or P depletion. Properly managed scenario: P fertilizer and lime are applied when AP or pH fall below optimal levels (Figure S3h,k, Supporting Information), allowing rice yield enhancement to continuously benefit from soil health improvements.

Our results provide direct evidence that the CO_2_ fertilization effect in rice paddies has persisted after more than a decade of eCO_2_ exposure (Figure 4a,b), with no detectable reduction in rice N or P uptake (Figure S6, Supporting Information). Moreover, improvements in soil health contributed to sustaining the CO_2_ fertilization effect (Figure 4c), and overlooking this indirect benefit may lead to an underestimation of rice yields in future climate scenarios (**Figure** **5**).

Over the past few decades, the CO_2_ fertilization effect has declined globally, except in certain croplands of Southeast Asia, eastern Australia, and North America.^[^ ^7^ ^]^ Evidence from eCO_2_ experiments further suggests that productivity gains in natural ecosystems are likely to plateau or even reverse in the future due to edaphic stressors^[^ ^49^, ^50^ ^]^ particularly N^[^ ^5^, ^48^ ^]^ and P^[^ ^6^, ^34^, ^51^ ^]^ limitations. Compounding this, initial increases in productivity amplify plant nutrient demand, accelerating depletion of soil nutrient pools and widening existing nutrient gaps.^[^ ^5^, ^7^ ^]^ Long‐term eCO_2_ generally does not exacerbate soil N limitation, due to natural biological MN supply (Figures S9e,S21, Supporting Information).^[^ ^9^, ^10^ ^]^ By contrast, P depletion intensifies over prolonged eCO_2_ exposure (Figure S9g, Supporting Information). In natural ecosystems where microbial growth is predominantly P‐limited,^[^ ^52^ ^]^ the additional plant‐derived C under eCO_2_ heightens microbial competition with plants for P, further constraining the long‐term persistence of the CO_2_ fertilization effect.^[^ ^6^, ^34^ ^]^

Croplands, however, differ fundamentally from natural ecosystems. Frequent inorganic fertilization maintains higher nutrient availability and shifts microbial metabolism toward a C‐limited regime, favoring P mineralization over assimilation.^[^ ^35^, ^53^ ^]^ As a result, both paddy and upland soils experience slower P depletion than non‐cropland soils (Figure S22, Supporting Information), lowering the risk of P limitation and the associated attenuation of CO_2_ fertilization. The two FACE sites studied here are representative of Chinese rice paddies in terms of nutrient supply (Figure S2, Supporting Information). Under such conditions, the gradual decline in AP over decades is unlikely to cause agronomic P deficiency (Figure 1a; Figure S5, Supporting Information);^[^ ^12^ ^]^ instead, it likely curbs P losses and mitigates water pollution.^[^ ^12^, ^19^ ^]^ Thus, rice paddies in P‐rich regions are well positioned to benefit from eCO_2_ with minimal environmental burden. Conversely, in P‐deficient areas, strategic P fertilization remains essential to balance yield gains with reduced P pollution risk.^[^ ^12^ ^]^

Meanwhile, eCO_2_‐induced acidification may pose a long‐term constraint on soil health (Figure S9a, Supporting Information). Proactive measures, such as liming, should therefore be implemented to stabilize soil pH. This strategy not only curbs further acidification but also enhances crop yields and reduces GHG emissions,^[^ ^1^, ^54^ ^]^ thereby offering co‐benefits for food security and climate mitigation. By adopting such practices, improvements in soil health can be fully leveraged to sustain ecosystem services in rice paddies under long‐term eCO_2_ exposure.

## 4 Conclusion

In summary, our long‐term FACE experiments provide novel, comprehensive evidence that eCO_2_ enhances paddy soil health, improving soil fertility and reinforcing the CO_2_ fertilization effect. Beyond sustaining rice productivity, these gains position rice paddies as strategic allies in climate change mitigation and water quality regulation. Given the persistent inadequacy of current mitigation efforts,^[^ ^55^ ^]^ the positive response of rice paddies to eCO_2_ offers a timely opportunity to advance the transition toward a sustainable, climate‐resilient rice production system. Looking ahead, governments should prioritize interventions to prevent soil acidification in rice paddies, while strengthening international cooperation to support low‐income regions in tackling long‐standing P deficiencies exacerbated by eCO_2_.^[^ ^12^, ^56^ ^]^ Such coordinated, equitable action will ensure that enhanced soil health continues to underpin global food security in the face of climate change challenges.

## 5 Experimental Section

### The Field Rice FACE Experiments

Study NP_M_ was initiated in June 2004 at Zongcun Village, Jiangdu District, Yangzhou City, Jiangsu Province, China (32° 35′ N, 119° 42′ E; Figure S1a, Supporting Information). This region features a northern subtropical monsoon climate with an average annual precipitation of 1000 mm, an average annual temperature of 15 °C, and an average annual frost‐free period of 220 days. The FACE system consisted of three ambient CO_2_ (aCO_2_) rings and three FACE rings with elevated CO_2_ (200 µmol·mol^−1^ above the ambient, eCO_2_; Supplementary Information). The soil, classified as Shajiang‐Aquic Cambisol, contains 13.7% clay, 28.5% silt, and 57.8% sand, with moderate N and P content (Figure S2, Supporting Information), hence coded as NP_M_ in this study. Before CO_2_ fumigation began in 2004, the soil had a pH of 6.8, SOC content of 1.84%, TN content of 1.45 g·kg^−1^, TP content of 0.63 g·kg^−1^, and a bulk density of 1.16 g·cm^−3^. Rice seeds (*Oryza sativa* cv.) were sown from mid to late May, with seedlings transplanted each year in mid to late June. More details on crop cultivation and fertilizer application can be found in the previous studies.^[^ ^12^, ^25^ ^]^

Study NP_H_ commenced in April 2011 at Kangbo Village, Changshu City, Jiangsu Province, China (31° 30′ N, 120° 33′ E; Figure S1b, Supporting Information). The climate is similar to that of the NP_M_ site, with an average annual precipitation of 1100 mm, an average annual temperature of 16 °C, and a frost‐free period of 220 days. This FACE system also included three control rings with aCO_2_ and three FACE rings with eCO_2_ (100 µmol·mol^−1^ above ambient during 2011–2018, and 200 µmol·mol^−1^ above ambient during 2019–2022; Supplementary Information). The soil, classified as a Gleyic‐Stagnic Anthrosol, contains 8.2% clay, 78.5% silt, and 13.3% sand, with high N and P content (Figure S2, Supporting Information), hence coded as NP_H_ in this study. Prior to CO_2_ fumigation in 2011, the soil had a pH of 7.0, SOC content of 1.60%, TN content of 1.90 g·kg^−1^, TP content of 0.90 g·kg^−1^, and a bulk density of 1.10 g·cm^−3^. Rice and winter wheat were rotated at this site according to local agronomic practices. Further details on crop cultivation and fertilizer application can be found in the previous studies.^[^ ^12^, ^57^ ^]^

### Soil and Plant Sampling

Soil samples were collected at the rice ripening stage in 2015 (year 12) and 2018 (year 15) in study NP_M_, and in 2022 (year 12) in study NP_H_. In 2015 (NP_M_) and 2022 (NP_H_), five soil cores (2.5 cm diameter × 20 cm deep) were collected and composited into a single sample per ring. The NP_M_ study employed a split‐plot design, with CO_2_ as the main plot factor and rice cultivar as the split‐plot factor. To account for varietal differences in rice responses to eCO_2_,^[^ ^24^, ^25^ ^]^ each ring was divided into three sub‐plots in 2018 according to previous planting history (*Japonica*, *Indica*, and mixed). Five soil cores were randomly collected from each sub‐plot and combined into a composite sample, yielding 18 samples—9 for aCO_2_ and 9 for eCO_2_. After soil sampling, part of the fresh soil was immediately frozen in liquid nitrogen and subsequently stored at −80 °C for biological analysis. The remaining soil was air‐dried, ground, passed through a 2 mm sieve, and stored at room temperature for chemical analysis. Rice grains were harvested from NP_M_ (2015, 2017, and 2018) and NP_H_ (2019–2022), all derived from Wunyunjing 23, a conventional *Japonica* rice variety widely cultivated in Jiangsu Province, China. The frequency of measurement and sample batches used for each indicator is shown in Table S4 (Supporting Information).

### Chemical Analysis

SOC was measured using the potassium dichromate volumetric method.^[^ ^14^ ^]^ TN was determined by the semi‐micro Kjeldahl method.^[^ ^58^ ^]^ TP was determined by inductively coupled plasma optical emission spectrometer (ICP‐OES) after aqua regia digestion.^[^ ^6^ ^]^ TK was determined by ICP‐OES after nitric acid‐perchloric acid‐hydrofluoric acid digestion. MN (the sum of NH_4_⁺ and NO_3_^−^) was extracted using 2 M KCl (1:5 soil/water) and then determined by a continuous flow analyzer.^[^ ^24^ ^]^ AP was extracted with anion exchange resin and 0.5 M NaHCO_3_, then determined using the ascorbic acid molybdenum blue method (i.e., the sum of resin‐P and NaHCO_3_ inorganic P defined in the modified Hedley method).^[^ ^6^, ^12^ ^]^ AK was extracted using 1 M ammonium acetate and then determined by ICP‐OES.^[^ ^14^ ^]^ pH was measured with a glass electrode in a 1:2.5 soil/water suspension. EC was measured with a conductivity meter in the supernatant after leaching in deionized water (1:5 soil/water). CEC was measured by the hexamminecobalt trichloride solution‐spectrophotometric method. Grain N content was determined by the Kjeldahl method.^[^ ^59^ ^]^ Grain P content was digested with sulfuric acid and hydrogen peroxide and then determined by ICP‐OES.^[^ ^12^ ^]^

### Biological Analysis

MBC was determined using the chloroform fumigation–extraction method.^[^ ^45^ ^]^ For the determination of AMF biomass, soil phospholipid fatty acids were extracted from freeze‐dried samples with a single‐phase mixture of chloroform, methanol, and citrate buffer (1:2:0.8, v/v/v; 0.15 M, pH 4.0). The resulting fatty acid methyl esters were separated and identified using an Agilent 7890N gas chromatograph equipped with a MIDI Sherlock microbial identification system (version 4.5). The fatty acids 18:1ω9c and 18:2ω6,9c were used as fungal biomarkers, whereas 16:1ω5c was used as the AMF biomarker.^[^ ^12^, ^25^ ^]^ The procedures for fungal internal transcribed spacer (ITS) sequencing and metabolomics analysis are described in the Supplementary Information.

### Soil Health Assessment

The concept of soil health has evolved from soil quality and continues to develop, making it difficult to clearly distinguish between them due to their overlapping definitions.^[^ ^18^, ^26^ ^]^ Compared to soil quality, soil health adopts a broader perspective, encompassing the full spectrum of soil ecosystem services rather than focusing solely on plant growth.^[^ ^16^, ^18^ ^]^ However, in rice paddies, the primary ecosystem service is food production, and thus the terms “soil health” and “soil quality” are used interchangeably in this study.

It is important to note that soil property measurements do not always correlate linearly with ecosystem functions. Three common scenarios can generally be observed:^[^ ^15^, ^17^ ^]^ (i) soil function increases with higher measurements (e.g., SOC content and soil C sequestration capacity); (ii) soil function decreases with higher measurements (e.g., soil hardness and roots penetration capacity); and (iii) soil function responds nonlinearly to increasing measurements (e.g., pH and plant productivity, where both increases and decreases beyond the optimal pH range can negatively affect plant growth). Thus, converting soil property measurements into soil health scores requires establishing reasonable and applicable standards.^[^ ^18^ ^]^

In this study, soil health was assessed using the widely adopted CASH protocol (https://soilhealthlab.cals.cornell.edu/).^[^ ^15^, ^26^, ^60^ ^]^ This framework provides a standardized yet adaptable approach, incorporating baseline soil properties representative of a single land‐use type within a defined geographic area, and allows the development of site‐specific scoring curves that translate measured values into soil health scores.^[^ ^17^ ^]^ By integrating local context, CASH enhances both the interpretability and environmental relevance of soil health assessments,^[^ ^18^ ^]^ supporting evidence‐based land management through individual indicator scores.^[^ ^26^, ^60^ ^]^ This approach also enables to discern how eCO_2_ specifically influences soil health, rather than merely providing broad directional conclusions.

Based on the CASH protocol and previous studies,^[^ ^15^, ^16^, ^17^, ^18^, ^20^, ^21^, ^26^, ^60^, ^61^ ^]^ soil properties related to three key components of soil health—food production, water quality control, and climate change mitigation—were selected for assessment. These properties (SOC, TN, TP, TK, MN, AP, AK, MBC, pH, CEC, and EC; Table S1, Supporting Information) collectively provide a comprehensive perspective on the climate resilience of rice paddies. Physical indicators, such as soil penetration resistance, were not included in this study because of the intensive tillage practices at the experimental sites. Nevertheless, physical indicators remain essential for evaluating soil health in ecosystems with minimal human disturbance.^[^ ^18^ ^]^

Field observations for all indicators were obtained from Chinese rice paddies, except for MBC (see below). Data for SOC, pH, AP, and AK were obtained from the National Soil Test and Fertilizer Recommendation Projects.^[^ ^14^, ^21^ ^]^ Data for TP were obtained from a nationwide analysis of soil P balance conducted in China.^[^ ^19^ ^]^ Data for TN, MN, TK, CEC, and EC were compiled from a synthesis of published literature retrieved from the Web of Science online database (WOS, https://webofscience.clarivate.cn/) using the keywords “China” AND “soil” AND “Paddy” AND “Field experiment” (Data S3, Supporting Information). However, as MBC is not routinely measured as a fundamental soil property, few measurements were available in the compiled studies. Therefore, global paddy soil data (ref. [31]) were used as a substitute. This substitution is unlikely to bias the results substantially for two reasons: first, a large portion of the global MBC dataset originates from China;^[^ ^31^ ^]^ second, the MBC indicator score increases monotonically with measurement values, so minor variations in the scoring curve do not affect the overall trend (Figure S3f, Supporting Information). Also, note that the method used to measure AP better reflects the pool of P accessible to plants,^[^ ^6^, ^12^ ^]^ yet the National Soil Test and Fertilizer Recommendation Projects report Olsen‐P for convenience. To ensure consistency, AP values were converted using the established relationship with Olsen‐P in rice paddies (AP = 1.04 × Olsen‐P + 9.08; ref.[12]).

According to the CASH protocol, the scoring curves were categorized as “more is better” and “optimal is better” (Figure S3, Supporting Information). The “more is better” category, which includes SOC, TN, TP, TK, CEC, and MBC, was fitted with the Boltzmann curve, reflecting a positive effect with increasing values. The “optimal is better” category, which includes MN, AP, AK, EC, and pH, was fitted with the Gaussian curve, so that any increase or decrease in values deviating from the optimum negatively affects soil health. Outliers in the observation data were identified using the Grubbs test (threshold: *p* = 0.05) and removed. The data were then divided into intervals based on the dataset size, with small datasets (*n* < 2000) divided into 15 equally spaced intervals, medium datasets (2000 ≤ *n* ≤ 5000) divided into 50 intervals, and large datasets (*n* > 5000) divided into 100 intervals. Soil health index was calculated as the unweighted average of individual indicator scores. This approach is appropriate because, although some indicators overlap in the ecosystem services they reflect, each emphasizes different aspects (Table S1, Supporting Information),^[^ ^18^ ^]^ and the CASH protocol accordingly relies on individual indicator scores to inform land management decisions.^[^ ^60^ ^]^

### Systematic Synthesis of Soil Health Responses to eCO_2_

In September 2025, the WOS database was searched using the query: (“rising” OR “elevated” OR “increased” OR “enrichment”) AND “CO_2_” AND “soil”. Our aim was to compile published responses to eCO_2_ for seven key soil health indicators—pH, SOC, TN, TP, MBC, MN, and AP—defined as properties showing significant responses in at least one of three sampling periods across the two study sites (Figure 1a). Study selection and screening followed the Preferred Reporting Items for Systematic Reviews and Meta‐Analyses (PRISMA) framework (Figure S7, Supporting Information).

The collected studies (Data S1, Supporting Information) covered four major ecosystem types: rice paddies, upland croplands, forests, and grasslands (including shrublands and tundra). Data from field‐based rice eCO_2_ experiments conducted in China were extracted from the compiled dataset and further evaluated using the soil health assessment framework to examine the generality of eCO_2_ effects on paddy soil health (Data S2, Supporting Information).

Most of the included observations were derived from FACE systems or open‐top chambers (OTCs), which best simulate realistic climate change conditions.^[^ ^2^, ^27^ ^]^ A small subset of studies (5 of 253) with comparable experimental designs was also retained (see Supplementary Information). Data were obtained directly from texts, tables, and supplementary materials, or digitized from figures using GetData Graph Digitizer version 2.20. Additional details on data extraction are provided in the Supplementary Information

For each study, both the natural logarithm of the response ratio (RR = eCO_2_ / aCO_2_) and the proportional effect size ((eCO_2_ − aCO_2_) / aCO_2_) were calculated to quantify the impacts of eCO_2_ on soil health indicators.^[^ ^9^, ^27^, ^62^ ^]^ Confidence intervals were estimated by bootstrap resampling (1000 iterations), and effects were considered significant when the 95% confidence interval did not include zero.^[^ ^6^ ^]^

### Statistical Analysis

Significance tests and correlation analysis were carried out using SPSS Statistics 26.0 (IBM, USA). For comparisons between two groups, the Student's *t*‐test was used for normally distributed data, and the Mann–Whitney U test otherwise. One‐way analysis of variance (ANOVA) was applied for comparisons among more than two groups. All tests were two‐sided, with significance defined as *p* < 0.05 and marginal significance as 0.05 ≤ *p* < 0.10. GLMs were used to assess the effects of eCO_2_ on soil health indicator scores, with CO_2_ treatment and site included as fixed factors and a Gamma distribution with a log link specified for the models;^[^ ^63^ ^]^ robust HC3 standard errors were applied, and *p*‐values were adjusted for multiple comparisons using the false discovery rate (FDR) method.

Fungal *α* diversity, principal coordinate analysis (PcoA), and PERMANOVA were conducted using the “Vegan” package.^[^ ^64^ ^]^ To evaluate overall differences in fungal diversity, each metric (from left to right in Figure 3b: observed species, Chao1 index, ACE index, Simpson index, Pielou's evenness, and Shannon index) was *z*‐standardized and combined to avoid dominance by any single indicator. Nutrient limitation was estimated as the average of the inverse *z*‐scores of SOC, TN, and TP. Hierarchical partitioning quantified the contribution of nutrient supply to variation in interspecific relationships.^[^ ^63^ ^]^ Biomarkers sensitive to eCO_2_ were identified through Linear Discriminant Analysis Effect Size (LEfSe) via Galaxy (http://huttenhower.sph.harvard.edu/galaxy/), while co‐occurrence networks were visualized in Gephi (version 0.9.2). Abundance‐weighted, null model–corrected fungal interspecific competition strength was quantified by negative cohesion.^[^ ^47^, ^65^ ^]^ Predicted ecological functions of fungi were inferred using FUNGuild, excluding annotations with a confidence ranking of “possible”.^[^ ^66^ ^]^ Welch's *t*‐test in STAMP was employed to identify sensitive fungal guilds.^[^ ^67^ ^]^

Causal mediation analysis was conducted using the “mediation” package,^[^ ^68^ ^]^ with confidence intervals estimated via 1000 nonparametric bootstraps. Differences in metabolomic profiles were analyzed by orthogonal partial least squares–discriminant analysis (OPLS‐DA) in MetaboAnalyst (http://www.metaboanalyst.ca/), with differentially expressed metabolites (DEMs) defined as VIP > 1 and *p* < 0.05. Metabolites were classified by ChEBI Ontology (https://www.ebi.ac.uk/chebi/), and perturbed biological pathways were analyzed using MBROLE 2.0.^[^ ^69^ ^]^ Structural equation modeling (SEM) with robust maximum likelihood estimation was performed in SPSS AMOS 22 (IBM, USA; Supplementary Information).

## Conflict of Interest

The authors declare no conflict of interest.

## Author Contributions

C.Z., H.G., and W.D. performed conceptualization; C.Z., H.G., W.D., F.J., L.S., C.C., and Y.W. performed methodology; F.J., W.D., L.D., Z.H., W.S., Q.W., H.Y., Y.Y., and F.A. performed investigation; C.Z., W.D., and F.J. performed data curation; F.J., W.D., K.J.v.G., J.P., and S.X.C. performed formal analysis; F.J., W.D., K.J.v.G., J.P., S.X.C., and C.Z. wrote original draft; F.J., W.D., K.J.v.G., J.P., S.X.C., W.Z., D.W., J.S., X.Y., R.S., J.Z., H.G., and C.Z. wrote, reviewed, and edited the manuscript; C.Z. and W.D. performed supervision.

## Supporting information



Supporting Information

Supporting Information

## Data Availability

The data that support the findings of this study are openly available in figshare at https://doi.org/10.6084/m9.figshare.27636876, reference number 27636876.
