# Supplementary material for: Long‐Term Elevated CO2 Improves Soil Health and Rice Yields in Paddy Fields
Source: Adv Sci (Weinh). 2025 Dec 7;13(11):e03190. doi: 10.1002/advs.202503190 (PMC12931181; doi:10.1002/advs.202503190)
Supplement: Supplementary file 1 — Supporting Information [file ADVS-13-e03190-s002.docx]

Supporting Information for

Long-term elevated CO₂ improves soil health and rice yields in paddy fields

Fan Jiang, Wenchao Du*, Kees Jan van Groenigen, Josep Peñuelas, Scott X. Chang, Lian Song, Chuang Cai, Yu Wang, Lin Deng, Zhengkun Hu, Weishou Shen, Qicong Wu, Haiyang Yu, Ying Yin, Fuxun Ai, Wei Zhou, Dongming Wang, Jianqiang Sun, Xiaoyuan Yan, Renfang Shen, Jiabao Zhang, Hongyan Guo, Chunwu Zhu*

E-mail: cwzhu@issas.ac.cn (C. Z.); du@njnu.edu.cn (W. D.)

**This file includes:**

Supplemental Figures S1–S22

Supplemental Tables S1–S4

Supplemental Methods

1. **Supplemental Figures**

Figure S1. Aerial views of the two field rice FACE experiments. (a) Jiangdu FACE. (b) Changshu FACE. Both sites are located near the Yangtze River in China. Jiangdu FACE (study NP_M_) had moderate total N and P levels, whereas Changshu FACE (study NP_H_) had high total N and P levels (Figure S2). Details on the experimental design and operation are provided in the Experimental section and Supplemental Methods.

Figure S2. Soil nutrient status across Chinese rice paddies and the relative positions of the two FACE sites. (a) Density distribution of total N in the topsoil of Chinese rice paddies. (b) Density distribution of total P in the topsoil of Chinese rice paddies. Density distributions were derived from observational data on Chinese rice paddies (Data S3 and ref. ^[1]^). The Jiangdu FACE site (black solid line) represents medium N and P levels (NP_M_), while the Changshu FACE site (black dashed line) represents high N and P levels (NP_H_).

Figure S3. Scoring curves for soil health indicators. The curves were developed from observational data on Chinese rice paddies (Experimental Section; Data S3 and refs. ^[1-4]^). Extreme fitted values may slightly exceed the 0–100 range; any calculated scores below 0 should be set to 0, and those above 100 should be set to 100.

Figure S4. Supplemental indicators for soil health assessment. TK, total K; AK, available K; CEC, cation exchange capacity; EC, electrical conductivity. Data are shown as mean ± standard deviation (n = 9 for NP_M_ 2018; n = 3 for NP_H_ 2022).

Figure S5. Estimation of soil P deficiency thresholds in two Chinese rice FACE experiments. (a) Conceptual model of major P fluxes. (b) Estimation of soil P deficiency thresholds. In our study sites, the major P outputs included aboveground harvest (grain and straw) and water erosion/leaching, whereas the only P input was fertilizer application. The following assumptions were applied: 1) all P output originates from the available P pool because this is the most active part of soil P stock;^[1,5]^ 2) all P input enters the available P pool, as only inorganic fertilizers were used;^[6]^ 3) rice P uptake occurs solely within the plow layer (0–20 cm). The P gap (i.e., P demand exceeding fertilizer input) was calculated as P output minus P input. Data on harvested P were obtained from our previous study.^[5]^ P loss from water erosion/leaching was estimated as 82% of the harvested P, representing the upper limit for Chinese paddies.^[1]^ P deficiency thresholds were determined using soil bulk density and the calculated P gap. Because P outputs were likely overestimated and inputs underestimated, the derived thresholds represent conservative (upper-bound) estimates of the P supply required to sustain rice growth.

Figure S6. Rice nutrient response to eCO₂. (a) N and P content and total N and P harvested in rice grain in the study NP_M_. (b) N and P content and total N and P harvested in rice grain in the study NP_H_. Data are shown as mean ± standard deviation (n = 3). Asterisks and plus signs indicate significant (*p* < 0.05) and marginally significant (*p* < 0.1) differences, respectively, based on the Student’s t-test.

Figure S7. PRISMA flow diagram showing the study selection procedure for the data synthesis.

Figure S8. Temporal trends in the response of key soil health indicator scores in Chinese rice paddies under eCO₂. Results are synthesized from published field-based eCO₂ experiments conducted in Chinese rice paddies (Data S2). SOC, TN, and MBC were identified as key indicators based on our two FACE experiments (Figure 1b). Due to the limited dataset and incomplete records across indicators, data were grouped into three intervals of eCO₂ exposure duration. The mean response ratio within each interval was used to represent the average temporal trend of each indicator. One-sample t-tests were used to determine whether response ratios significantly differed from zero (no-effect value).

Figure S9. Responses of soil health indicators to eCO₂ across terrestrial ecosystems. Results are based on this study and peer-reviewed publications (Data S1). Data with exposure durations shorter than one year were excluded from regression to ensure that steady-state responses were captured.^[7]^ The shaded area denotes the 95% confidence interval of the fitted line, and the black dashed line indicates the no-effect value.

Figure S10. Role of microbial P limitation in the response of total P to eCO₂. Results are based on this study and peer-reviewed publications (Data S1). The black dashed line indicates the no-effect value. P limitation thresholds were defined according to microbial stoichiometric (C/P = 42) and metabolic (C/P = 126) requirements,^[8]^ distinguishing no (C/P < 42), weak (42 ≤ C/P ≤ 126), and strong (C/P > 126) P limitation. In (a), the shaded area represents the 95% confidence interval of the fitted line. The C/P ratio was calculated as the mean under aCO₂ and eCO₂ conditions. In (b), circles show mean values of raw data, and bars represent 95%, 85%, and 75% bootstrap confidence intervals (lighter colors indicate higher confidence).

Figure S11. Fungal biomarkers responding to eCO₂. (a) Fungal taxa significantly affected by eCO₂ at the class, order, family, genus, and species level, identified by Welch’s t-test. (b, c) Biomarkers identified by linear discriminant analysis effect size (LEfSe). (d) Functional roles of fungal biomarkers summarized from refs. ^[9-20]^. Soil samples for fungal community characterization were collected from study NP_M_ in 2018 (n = 9).

Figure S12. Functional responses of soil fungi to eCO₂. (a) Sensitive guilds identified by FUNGuild. (b) Fungal trophic modes identified by FUNGuild. Data are shown as mean ± standard deviation (n = 9). Soil samples were collected from study NP_M_ in 2018. In (a), sensitive guilds were identified by Welch’s t-test (*p* < 0.05). In (b), the sum of trophic modes exceeds 100% because many fungi exhibit multiple trophic strategies; significance was tested by the Student’s t-test.

Figure S13. Responses of soil metabolic profiles to eCO₂. (a) Cluster heatmap of differentially expressed metabolites (DEMs). (b) Classification of down-regulated DEMs. (c) Classification of up-regulated DEMs. Soil samples were collected from study NP_M_ in 2018 (n = 9). DEMs were identified using orthogonal partial least squares–discriminant analysis (OPLS-DA) with VIP > 1 and *p* < 0.05. In (a), A1–A9 and F1–F9 denote the 9 samples collected from aCO₂ and eCO₂ plots, respectively. In (b) and (c), up-regulation indicates enrichment under eCO₂ and vice versa.

Figure S14. Fit of the neutral community model (NCM) for fungal community assembly. *R*^2^ indicates model fit, and *m* represents migration rate. Solid lines denote optimal model fits; dashed lines indicate the 95% confidence boundary. OTUs occurring more (green) or less (orange) frequently than predicted are highlighted relative to those matching the model (blue).

Figure S15. Relationship between soil health index and rice yield. Data for the fitting were obtained from Figure 1c and Figure 4a, with the shaded area indicating the 95% confidence interval of the fitted line.

Figure S16. *A priori* conceptual model used for structural equation modeling (SEM). The model explores the mechanisms linking altered C, N, and P biogeochemical cycling under eCO₂. Arrows indicate hypothesized causal pathways; and numbers denote interaction types: 1) eCO₂ effects on soil C and nutrient pools via changes in plant residue and rhizodeposits; 2) microbial mineralization–assimilation trade-offs reshaping soil N and P pools under eCO₂; and 3) soil bioavailable N and P supply governing by mineralization–assimilation dynamics. Paths marked with red crosses were excluded from the final model due to weak and non-significant coefficients (absolute value < 0.05, *p* > 0.10).

Figure S17. SEM uncovering potential causal relationships among soil C, N, and P dynamics under eCO₂. (a) Path diagram. (b) Summed direct and indirect effects. The model was constructed based on the *a priori* model (Figure S16) using data from study NP_M_. In (a), arrows represent causal pathways, and numbers indicate standardized path coefficients (analogous to regression weights). *R*^2^ denotes the proportion of variance explained by the model. Orange and blue arrows correspond to positive and negative relationships, respectively. Only significant (solid line, *p* < 0.05) and marginally significant (dashed line, *p* < 0.1) paths are shown. Model fit indices indicate a good fit: Chi-square = 6.033, *p* = 0.420, df = 6, GFI = 0.938, CFI = 1.000, RMSEA = 0.015.

Figure S18. Contrasting dynamics of soil N and P pools under eCO₂. (a) Decoupled P–C dynamics. (b) Coupled N–C dynamics. Results are based on this study and peer-reviewed publications (Data S1). Shaded areas denote 95% confidence intervals.

Figure S19. Differential responses of MBC to eCO₂ in croplands and natural ecosystems. Results are based on this study and peer-reviewed publications (Data S1). Black circles and error bars indicate mean ± 95% confidence interval, and significance was assessed using the Mann–Whitney U test.

Figure S20. Potentially perturbed pathways under eCO₂. Soil samples were collected in 2018 from study NP_M_. DEMs were mapped onto the Kyoto Encyclopedia of Genes and Genomes (KEGG) database to identify significantly perturbed pathways.

Figure S21. Effects of long-term eCO₂ on soil N mineralization capacity. N mineralization was determined via an 88-day waterlogged incubation (Supplemental Methods). Soil samples were collected in 2015 from study NP_M_. Asterisks indicate significant differences based on the Student’s t-test (**p* < 0.05; ***p* < 0.01; and ****p* < 0.001).

Figure S22. Differential responses of available P to eCO₂ in croplands and natural ecosystems. Results are based on this study and peer-reviewed publications (Data S1). Black circles and error bars indicate mean ± 95% confidence interval, and significance was assessed using the Mann–Whitney U test.

2. **Supplemental Tables**

Table S1 Soil health indicators and their relevance to ecosystem services of rice paddies.

| Indicators | Ecosystem services | | |
| --- | --- | --- | --- |
|  | Plant production | Water quality control | Climate change mitigation |
| SOC | ○ | ○ | ○ |
| TN | ○ | × | × |
| TP | ○ | × | × |
| TK | ○ | × | × |
| CEC | ○ | ○ | × |
| MBC | ○ | ○ | ○ |
| MN | ○ | ○ | ○ |
| AP | ○ | ○ | ○ |
| AK | ○ | ○ | ○ |
| EC | ○ | ○ | × |
| pH | ○ | ○ | ○ |

SOC: soil organic carbon; TN: total nitrogen; TP: total phosphorus; TK: total potassium; MN: mineral nitrogen; AP: available phosphorus; AK: available potassium; MBC: microbial biomass carbon; CEC: cation exchange capacity; EC: electrical conductivity. Indicators less relevant to a given ecosystem service are denoted by “×”, and those more relevant by “○”. The classification is based on refs. ^[3,21-28]^.

Table S2 Topological properties of co-occurrence networks within fungal genera.

| Network topological properties | Ambient | FACE |
| --- | --- | --- |
| Number of nodes | 83 | 83 |
| Number of edges | 118 | 136 |
| Positive correlation (%) | 55.1 | 71.3 |
| Negative correlation (%) | 44.9 | 28.7 |
| Modularity | 0.733 | 0.742 |
| Average clustering coefficient | 0.366 | 0.577 |
| Average path length | 4.739 | 4.951 |
| Network diameter | 12 | 11 |
| Graph density | 0.035 | 0.04 |
| Average degree | 2.843 | 3.277 |

Table S3 Relationship between individual indicators of soil health and rice yield.

| Indicators | Measurements | | Indicator scores | |
| --- | --- | --- | --- | --- |
|  | *R^2^* | *p*-value | *R^2^* | *p*-value |
| SOC | 0.010 | 0.761 | 0.023 | 0.638 |
| TN | 0.071 | 0.402 | 0.059 | 0.449 |
| TP | 0.003 | 0.859 | 0.002 | 0.905 |
| TK | 0.187 | 0.161 | 0.188 | 0.159 |
| CEC | 0.058 | 0.449 | 0.057 | 0.456 |
| MBC | 0.142 | 0.228 | 0.144 | 0.224 |
| MN | 0.180 | 0.170 | 0.198 | 0.148 |
| AP | 0.267 | 0.085 | 0.174 | 0.177 |
| AK | 0.040 | 0.531 | 0.017 | 0.684 |
| EC | 0.000 | 0.969 | 0.065 | 0.423 |
| pH | 0.234 | 0.112 | 0.106 | 0.301 |

This table presents *R*^2^ values reflecting the strength of association between each individual indicator included in the index and rice yield. For the relationship between the soil health index and rice yield, see Figure S15.

Table S4 Measurement schedule of the indicators included in this study.

| Sample | Study site | Sampling year | Measured indicators |
| --- | --- | --- | --- |
| Soil | NP_M_ | 2015 | SOC, TN, TP, MN, AP, MBC, pH, PMN |
|  | NP_M_ | 2018 | SOC, TN, TP, TK, MN, AP, AK, MBC, CEC, EC, pH, fungal biomass, fungal community, metabolomics |
|  | NP_H_ | 2022 | SOC, TN, TP, TK, MN, AP, AK, MBC, CEC, EC, pH, fungal biomass |
| Plant | NP_M_ | 2015 | Rice yield, grain N content, grain P content |
|  | NP_M_ | 2017 | Rice yield |
|  | NP_M_ | 2018 | Rice yield, grain N content, grain P content |
|  | NP_H_ | 2019 | Rice yield |
|  | NP_H_ | 2020 | Rice yield, grain N content, grain P content |
|  | NP_H_ | 2021 | Rice yield |
|  | NP_H_ | 2022 | Rice yield, grain N content, grain P content |

PMN: potentially mineralizable nitrogen.

3. **Supplemental Methods**

**Details of the CO₂ fumigation in the field rice FACE experiments**

Both FACE experiments consisted of three plots under ambient atmospheric CO₂ (aCO₂) and three plots under elevated CO₂ (eCO₂), with all experimental plots arranged as octagonal rings—14 m in diameter for study NP_M_ and 8 m in diameter for study NP_H_. Under the intermediate greenhouse gas emissions scenario (SSP2-4.5), atmospheric CO₂ concentrations are projected to reach about 500 µmol·mol^⁻¹^ by 2050 and rise to 600 µmol·mol^⁻¹^ by the end of the century.^[29]^ Based on projected CO₂ concentration for 2050, the target CO₂ concentration for the eCO₂ plots in the NP_H_ study was set at 500 µmol·mol^⁻¹^ (100 µmol·mol^⁻¹^ above ambient) for the period 2011–2018. From 2019 onwards, in response to end-of-century scenarios, the target was increased to 600 µmol·mol^⁻¹^ (200 µmol·mol^⁻¹^ above ambient). Since its start in 2004, the NP_M_ study has maintained eCO₂ plots at 200 µmol·mol^⁻¹^ above ambient CO₂ levels for 15 years, until the facilities were closed after rice harvest in 2018.

In the eCO₂ plots, pure CO₂ is released to the center through peripheral emission tubes (variable in height to keep them above the rice canopy by 50 cm). CO₂ release was controlled by a computer program with an algorithm based on wind speed and direction to keep the target CO₂ concentration within the plot. In the rice-growing season in 2018, the average daytime CO₂ concentration in the eCO₂ plots in the study NP_M_ varied between 588 and 601 µmol·mol^⁻¹^ compared to 393 µmol·mol^⁻¹^ in the aCO₂ plots. The CO₂ concentrations at study NP_H_ were also satisfactorily controlled, with daytime CO₂ concentration averaging 580 µmol·mol^⁻¹^ in the eCO₂ plots and 397 µmol·mol^⁻¹^ in the aCO₂ plots in 2022. Before the onset of CO₂ fumigation, soil properties did not differ significantly among plots within each study site.

**Waterlogged incubation**

The waterlogged incubation followed the protocol described in our previous study.^[30]^ Briefly, 10 g of air-dried soil was placed in a 50 mL centrifuge tube and mixed with 20 mL of deionized water. The tubes were sealed and incubated in darkness at 30°C for 88 days in darkness. Soil leachates were collected on days 0, 4, 11, 18, 32, 46, 67, and 88 of the incubation. At each leaching, the contents were stirred for 30s and then centrifuged, and the supernatant was transferred to a 100 mL volumetric flask. The soils were then sequentially leached twice with 25 mL of 1 ᴍ KCl and twice with 15 mL of deionized water. All leachates were combined and brought to volume with deionized water, after which MN content (NH₄⁺ and NO₃⁻) was immediately determined using a continuous flow analyzer.

**Fungal community characterization**

For fungal internal transcribed spacer (ITS) sequencing, soil DNA was extracted using the DNeasy PowerSoil Kit (Qiagen, GER). Extracted DNA was diluted to 1 ng·μL^⁻¹^ and then used as the template for PCR amplification with universal primers ITS1F (5'-CTTGGTCATTTAGAGGAAGTAA-3') and ITS2 (5'-GCTGCGTTCTTCATCGATGC-3').^[31]^ After a second round of amplification and purification, equal amounts of final amplicon were pooled for sequencing on the Illumina MiSeq platform. Raw sequencing data were in FASTQ format. Paired-end reads were then preprocessed using Trimmomatic software to detect and cut off ambiguous bases.^[32]^ It also cut off low-quality sequences with an average quality score below 20 using a sliding window trimming approach. After trimming, paired-end reads were assembled using FLASH software.^[33]^ Parameters of assembly were: 10 bp of minimal overlapping, 200 bp of maximum overlapping, and 20% of maximum mismatch rate. Sequences were further processed using the QIIME software (version 1.8.0) to ensure accuracy by (i) excluding reads with ambiguous, homologous sequences or below 200 bp; and (ii) retaining reads with 75% of bases above Q20.^[34]^ Finally, UCHIME was used to detect and remove reads with chimera to obtain clean reads.^[35]^ Clean reads were subjected to primer sequences removal and clustering to generate operational taxonomic units (OTUs) using Vsearch software with a 97% similarity cutoff.^[36]^ The representative read of each OTU was selected using the QIIME package.^[34]^ All representative reads were identified and annotated against the Unite database (ITSs rDNA) using blast.^[37]^

**Soil metabolomics**

For non-targeted metabolomics analysis, equivalent fresh soil (1 g) was extracted with 1 mL of methanol: water (1:1 v/v) and then 20 μL of 2-chloro-l-phenylalanine (0.3 mg·mL^⁻¹^) dissolved in methanol was added as an internal standard. Two stainless steel balls were added to the extraction and pre-cooled at -20 °C followed by grinding at 60 Hz for 2 min. The sample was further homogenized by adding 2 mL of methanol: water (1:1 v/v), then centrifuged at 7700 rpm (4 °C) for 10 min. 2.5 mL of supernatant was taken and freeze-dried, then 400 μL of methanol: water (1:1 v/v) was added, and the sample was re-dissolved by vortex and sonication. The re-dissolved sample was centrifuged at 12000 rpm (4 °C) for 10 min, and 300 μL of the supernatant was placed in a glass sampling vial, then allowed to dry under vacuum at room temperature. After extraction of metabolites, 80 μL of methoxylamine hydrochloride (15 mg·mL^⁻¹^ in pyridine) was added to all samples, vortexed, and incubated at 37 °C for 90 min. Subsequently, 80 μL BSTFA (containing 1% TMCS) and 20 μL n-hexane were added to the mixture, vortexed, and then derivatized at 70 °C for 60 min. Before GC-MS analysis, the samples were left at room temperature for 30 min. The derivatized samples were analyzed on an Agilent 7890B gas chromatography system coupled to an Agilent 5977A MSD system. A DB-5MS fused silica capillary column (30 m × 0.25 mm × 0.25 μm) was utilized to separate the derivatives. Helium (> 99.999%) was used as the carrier gas at a constant flow rate of 1 mL·min^⁻¹^ through the column. The injector temperature was maintained at 260 °C. The injection volume was 1 μL by splitless mode. The initial oven temperature was 60 °C, ramped to 125 °C at a rate of 8 °C·min^⁻¹^, to 210 °C at a rate of 4 °C·min^⁻¹^, to 270 °C at a rate of 5 °C·min^⁻¹^, to 305 °C at a rate of 10 °C·min^⁻¹^, and finally held at 305 °C for 3 min. The temperature of the MS quadrupole and ion source (electron impact) was set as 150 and 230 °C, respectively. The quality control samples were injected at regular intervals throughout the analytical run to provide a set of data from which repeatability can be assessed. ChemStation (version E.02.02.1431) software was used to convert the raw data, followed by ChromaTOF software (version 4.34) for data processing. Metabolites were annotated through the Fiehn database. To get the final result, all internal standards and any known pseudo-positive peaks (caused by background noise, column bleed, or BSTFA derivatization procedure) were removed, and peaks from the same metabolite were combined.

**Data synthesis of soil health responses to eCO₂**

Five additional studies conducted in alternative experimental systems were also included in our synthesis, as these systems simulate climate change conditions in ways broadly comparable to FACE and OTC experiments. These include the Semi-open Air CO₂ Chamber (SACC), a semi-open climate simulation system positioned between FACE and OTC in design and function;^[38]^ Solardomes, which rely entirely on natural sunlight and maintain continuous air exchange with the external atmosphere through side windows and roof ventilation, thereby functioning similarly to OTCs;^[39,40]^ and Biosphere 2, a large-scale enclosed ecosystem that reconstructs integrated biogeochemical processes for long-term, whole-system experiments.^[41]^

During data extraction, several rules were applied to ensure consistency and minimize bias. All data represented topsoil physicochemical measurements, typically at 0–20 cm depth, though 0–5 cm, 0–10 cm, or 0–30 cm layers were accepted when standard depths were unavailable. If the topsoil was reported in separate sublayers, its physicochemical properties were averaged using bulk density-weighted means (where available) or thickness-weighted means. If both rhizosphere and bulk soil data were available, only rhizosphere values were used. Experimental duration was expressed in integer years. For natural ecosystems, durations were rounded to the nearest year, using six months as the threshold for rounding up. In croplands, single-season experiments were rounded similarly; the second season was recorded as two years, and each subsequent season was incremented by one year. To reduce pseudo-replication, only the final measurement was used for studies lasting less than one year, whereas for longer studies, measurements within the same year were averaged. When ambient CO₂ concentrations were unreported, a baseline of 400 µmol·mol^⁻¹^ was assumed.^[29]^ In multifactorial experiments involving additional treatments such as elevated temperature, O₃ enrichment, fertilization differences, or soil contamination, eCO₂ effects were assessed by keeping all other factors at the same level. For experiments in which CO₂ levels were gradually increased to a target, the effective enrichment level was calculated as the time-weighted average across all stages.

**Structural equation modeling**

Within the soil-plant continuum, eCO₂ initially enhances plant photosynthesis, which subsequently alters litter production and rhizodeposition, thereby modifying the inputs of C and other nutrients into the soil.^[42,43]^ These additional inputs alter microbial activity, shifting the balance between assimilation and mineralization, which in turn feeds back to influence the dynamics of soil C, N, and P.^[30,43]^ Accordingly, potential causal links among these elemental pools and their responses to eCO₂ were hypothesized, forming the basis of the *a priori* model used for structural equation modeling (SEM) analysis (Figure S16).

Suppose there are three variables (A, B, and C), where a change in A influences C indirectly through B. A basic principle in SEM is that the direct path from A to C should not be omitted unless there is clear evidence that A affects C exclusively via B. Following this principle, the remaining paths in the diagram (light grey) were specified. Only data from the NP_M_ site were used, as spatial heterogeneity among regions could otherwise bias estimates of eCO₂ effects. Prior to modeling, CO₂ concentrations were assigned as 400 for ambient rings and 600 for FACE rings,^[44]^ and all data were standardized using *z*-transformation (mean = 0, standard deviation = 1).^[45]^

**Supplemental References**

1	Xiaodong Song, Christine Alewell, Pasquale Borrelli *et al.* Pervasive soil phosphorus losses in terrestrial ecosystems in China. *Global Change Biology* **30**, e17108 (2024). https://doi.org/10.1111/gcb.17108

2	Liang Wei, Tida Ge, Zhenke Zhu *et al.* Paddy soils have a much higher microbial biomass content than upland soils: a review of the origin, mechanisms, and drivers. *Agriculture, Ecosystems & Environment* **326**, 107798 (2022). https://doi.org/10.1016/j.agee.2021.107798

3	Lei Qiao, Xuhui Wang, Pete Smith *et al.* Soil quality both increases crop production and improves resilience to climate change. *Nature Climate Change* **12**, 574-580 (2022). https://doi.org/10.1038/s41558-022-01376-8

4	Yuqing Ma, Dominic Woolf, Mingsheng Fan *et al.* Global crop production increase by soil organic carbon. *Nature Geoscience* **16**, 1159-1165 (2023). https://doi.org/10.1038/s41561-023-01302-3

5	Yu Wang, Yuanyuan Huang, Lian Song *et al.* Reduced phosphorus availability in paddy soils under atmospheric CO₂ enrichment. *Nature Geoscience* **16**, 162-168 (2023). https://doi.org/10.1038/s41561-022-01105-y

6	Xianzhen Luo, Ahmed S. Elrys, Lingling Zhang *et al.* The global fate of inorganic phosphorus fertilizers added to terrestrial ecosystems. *One Earth* **7**, 1402-1413 (2024). https://doi.org/10.1016/j.oneear.2024.07.002

7	Cesar Terrer, Richard P. Phillips, Bruce A. Hungate *et al.* A trade-off between plant and soil carbon storage under elevated CO₂. *Nature* **591**, 599-603 (2021). https://doi.org/10.1038/s41586-021-03306-8

8	Ji Liu, Tianyi Qiu, Josep Peñuelas *et al.* Crop residue return sustains global soil ecological stoichiometry balance. *Global Change Biology* **29**, 2203-2226 (2023). https://doi.org/10.1111/gcb.16584

9	Hexon Angel Contreras-Cornejo, Lourdes Macias-Rodriguez, Ek del-Val & John Larsen. Ecological functions of Trichoderma spp. and their secondary metabolites in the rhizosphere: interactions with plants. *FEMS Microbiology Ecology* **92**, fiw036 (2016). https://doi.org/10.1093/femsec/fiw036

10	Qiuling Dang, Yan Wang, Shangao Xiong *et al.* Untangling the response of fungal community structure, composition and function in soil aggregate fractions to food waste compost addition. *Science of the Total Environment* **769**, 145248 (2021). https://doi.org/10.1016/j.scitotenv.2021.145248

11	Sabrin R. M. Ibrahim, Shaimaa G. A. Mohamed, Ikhlas A. Sindi & Gamal A. Mohamed. Biologically active secondary metabolites and biotechnological applications of species of the family Chaetomiaceae (Sordariales): an updated review from 2016 to 2021. *Mycological Progress* **20**, 595-639 (2021). https://doi.org/10.1007/s11557-021-01704-w

12	Alfredo Justo, Alfredo Vizzini, Andrew M. Minnis *et al.* Phylogeny of the Pluteaceae (Agaricales, Basidiomycota): taxonomy and character evolution. *Fungal Biology* **115**, 1-20 (2011). https://doi.org/10.1016/j.funbio.2010.09.012

13	Kare Liimatainen, Jan T. Kim, Lisa Pokorny *et al.* Taming the beast: a revised classification of Cortinariaceae based on genomic data. *Fungal Diversity* **112**, 89-170 (2022). https://doi.org/10.1007/s13225-022-00499-9

14	Agostina V. Marano, Monica M. Steciow & Maria L. Arellano. New records of chytridiaceous fungi (Chytridiomycota) from the Reserva Natural Selva Marginal Punta Lara (Argentina) with comments on some previously reported species. *Nordic Journal of Botany* **26**, 248-254 (2008). https://doi.org/10.1111/j.1756-1051.2008.00214.x

15	François A. B. Olivier, Kyung W. Bang, Erica Zarate *et al.* Aerial warfare: an inducible production of volatile bioactive metabolites in a novel species of Scytinostroma sp. *Fungal Genetics and Biology* **158**, 103646 (2022). https://doi.org/10.1016/j.fgb.2021.103646

16	Shan-Ping Wan, Lan-Lan Huang, Meng-Jin Cui *et al.* Phylogenetic analyses of Hydnobolites and new species from China. *Journal of Fungi* **8**, 1302 (2022). https://doi.org/10.3390/jof8121302

17	Wenxiu Wang & Fengmao Chen. Role of the subtilisin-like serine protease CJPRB from Cordyceps javanica in eliciting an immune response in Hyphantria cunea. *International Journal of Molecular Sciences* **24**, 4170 (2023). https://doi.org/10.3390/ijms24044170

18	Zhi Xu, Liting Ma, Bing Zhao *et al.* Humification process enhancement through relative abundance promotion of Talaromyces and Coprinopsis by inoculated Phanerochaete chrysosporium during the secondary fermentation of composting. *Environmental Science and Pollution Research* **30**, 9060-9065 (2023). https://doi.org/10.1007/s11356-022-23846-4

19	Jing Yu, Jia Cai, Honggao Liu & Changlin Zhao. Molecular systematics and taxonomy reveal three new taxa in Phanerochaete (Phanerochaetaceae, Polyporales) from the Yunnan-Guizhou plateau, East Asia. *Mycological Progress* **22**, 59 (2023). https://doi.org/10.1007/s11557-023-01903-7

20	Ping Zhang, Li-Ping Tang, Qing Cai & Jian-Ping Xu. A review on the diversity, phylogeography and population genetics of Amanita mushrooms. *Mycology* **6**, 86-93 (2015). https://doi.org/10.1080/21501203.2015.1042536

21	Johannes Lehmann, Deborah A. Bossio, Ingrid Koegel-Knabner & Matthias C. Rillig. The concept and future prospects of soil health. *Nature Reviews Earth & Environment* **1**, 544-553 (2020). https://doi.org/10.1038/s43017-020-0080-8

22	Else K. Bunemann, Giulia Bongiorno, Zhanguo Bai *et al.* Soil quality-a critical review. *Soil Biology & Biochemistry* **120**, 105-125 (2018). https://doi.org/10.1016/j.soilbio.2018.01.030

23	Gerard H. Ros, Sven E. Verweij, Sander J. C. Janssen, Janjo De Haan & Yuki Fujita. An open soil health assessment framework facilitating sustainable soil management. *Environmental Science & Technology* **56**, 17375-17384 (2022). https://doi.org/10.1021/acs.est.2c04516

24	Jordon Wade, Steve W. Culman, Caley K. Gasch *et al.* Rigorous, empirical, and quantitative: a proposed pipeline for soil health assessments. *Soil Biology & Biochemistry* **170**, 108710 (2022). https://doi.org/10.1016/j.soilbio.2022.108710

25	Susan S. Andrews, Douglas L. Karlen & Cynthia A. Cambardella. The soil management assessment framework: a quantitative soil quality evaluation method. *Soil Science Society of America Journal* **68**, 1945-1962 (2004). https://doi.org/10.2136/sssaj2004.1945

26	Ferran Romero, Maeva Labouyrie, Alberto Orgiazzi *et al.* Soil health is associated with higher primary productivity across Europe. *Nature Ecology & Evolution* **8**, 1847-1855 (2024). https://doi.org/10.1038/s41559-024-02511-8

27	Kamal Nabiollahi, Fariba Golmohamadi, Ruhollah Taghizadeh-Mehrjardi, Ruth Kerry & Masoud Davari. Assessing the effects of slope gradient and land use change on soil quality degradation through digital mapping of soil quality indices and soil loss rate. *Geoderma* **318**, 16-28 (2018). https://doi.org/10.1016/j.geoderma.2017.12.024

28	Jialing Teng, Ruixing Hou, Jennifer A. J. Dungait *et al.* Conservation agriculture improves soil health and sustains crop yields after long-term warming. *Nature Communications* **15**, 8785 (2024). https://doi.org/10.1038/s41467-024-53169-6

29	IPCC. *Climate Change 2021: The Physical Science Basis*. (Cambridge University Press, 2021). https://doi.org/10.1017/9781009157896

30	Qicong Wu, Congzhi Zhang, Xuequan Liang *et al.* Elevated CO₂ improved soil nitrogen mineralization capacity of rice paddy. *Science of the Total Environment* **710**, 136438 (2020). https://doi.org/10.1016/j.scitotenv.2019.136438

31	Rachel I. Adams, Marzia Miletto, John W. Taylor & Thomas D. Bruns. Dispersal in microbes: fungi in indoor air are dominated by outdoor air and show dispersal limitation at short distances. *The ISME Journal* **7**, 1262-1273 (2013). https://doi.org/10.1038/ismej.2013.28

32	Anthony M. Bolger, Marc Lohse & Bjoern Usadel. Trimmomatic: a flexible trimmer for Illumina sequence data. *Bioinformatics* **30**, 2114-2120 (2014). https://doi.org/10.1093/bioinformatics/btu170

33	Deepak Reyon, Shengdar Q. Tsai, Cyd Khayter *et al.* FLASH assembly of TALENs for high-throughput genome editing. *Nature Biotechnology* **30**, 460-465 (2012). https://doi.org/10.1038/nbt.2170

34	J. Gregory Caporaso, Justin Kuczynski, Jesse Stombaugh *et al.* QIIME allows analysis of high-throughput community sequencing data. *Nature Methods* **7**, 335-336 (2010). https://doi.org/10.1038/nmeth.f.303

35	Robert C. Edgar, Brian J. Haas, Jose C. Clemente, Christopher Quince & Rob Knight. UCHIME improves sensitivity and speed of chimera detection. *Bioinformatics* **27**, 2194-2200 (2011). https://doi.org/10.1093/bioinformatics/btr381

36	Torbjorn Rognes, Tomas Flouri, Ben Nichols, Christopher Quince & Frederic Mahe. VSEARCH: a versatile open source tool for metagenomics. *PeerJ* **4**, e2584 (2016). https://doi.org/10.7717/peerj.2584

37	Stephen F. Altschul, Warren Gish, Webb Miller, Eugene W. Myers & David J. Lipman. Basic local alignment search tool. *Journal of Molecular Biology* **215**, 403-410 (1990). https://doi.org/10.1016/S0022-2836(05)80360-2

38	Pascal A. Niklaus, Ellen Kandeler, Paul W. Leadley *et al.* A link between plant diversity, elevated CO₂ and soil nitrate. *Oecologia* **127**, 540-548 (2001). https://doi.org/10.1007/s004420000612

39	James Heath, Edward Ayres, Malcolm Possell *et al.* Rising atmospheric CO₂ reduces sequestration of root-derived soil carbon. *Science* **309**, 1711-1713 (2005). https://doi.org/10.1126/science.1110700

40	Ladislav Holik, Valerie Vranova, Lenka Foltynova & Manuel Acosta. Forest soil properties under elevated CO₂: A five-year experiment. *European Journal of Soil Biology* **106**, 103346 (2021). https://doi.org/10.1016/j.ejsobi.2021.103346

41	Greg Barron-Gafford, Dean Martens, Katie Grieve *et al.* Growth of eastern cottonwoods (Populus deltoides) in elevated CO₂ stimulates stand-level respiration and rhizodeposition of carbohydrates, accelerates soil nutrient depletion, yet stimulates above- and belowground biomass production. *Global Change Biology* **11**, 1220-1233 (2005). https://doi.org/10.1111/j.1365-2486.2005.00985.x

42	Derek L. Sonderegger, Kiona Ogle, R. Dave Evans, Scot Ferguson & Robert S. Nowak. Temporal dynamics of fine roots under long-term exposure to elevated CO₂ in the Mojave Desert. *New Phytologist* **198**, 127-138 (2013). https://doi.org/10.1111/nph.12128

43	Jian Song, Shiqiang Wan, Shilong Piao *et al.* A meta-analysis of 1,119 manipulative experiments on terrestrial carbon-cycling responses to global change. *Nature Ecology & Evolution* **3**, 1309-1320 (2019). https://doi.org/10.1038/s41559-019-0958-3

44	ZhiBo Zhao, JiZheng He, Stefan Geisen *et al.* Protist communities are more sensitive to nitrogen fertilization than other microorganisms in diverse agricultural soils. *Microbiome* **7**, 33 (2019). https://doi.org/10.1186/s40168-019-0647-0

45	Travis S. Schmidt, Peter C. Van Metre & Daren M. Carlisle. Linking the agricultural landscape of the midwest to stream health with structural equation modeling. *Environmental Science & Technology* **53**, 452-462 (2019). https://doi.org/10.1021/acs.est.8b04381
